# Abstract of Meteorological Observations for January, 1854, Made at Philadelphia, Pa.

**Published:** 1854-03

**Authors:** James A. Kirkpatrick


*Abstract of Meteorological Observations for January,* 1854, *made at Philadelphia, Pa. By* PROF. JAMES A. KIRKPATRICK.

| 1854. Jan. | BAROMETER. Corrected for Temperature. Daily Mean. | Daily Range. | THERMOMETER. Fahrenheit. Mean. | Range | Dew point. 2 P. M. | Prevailing Winds. | General Remarks. Means made from the three Observations, viz: 7 A.M., 2 P. M., & 9 P. M. |
|---|---|---|---|---|---|---|---|
| | Inches. | Inch. | deg. | deg. | deg. | Points. | |
| 1 | 29.769 | .078 | 28.8 | 5 | 30.0 | S.W. | Morn. cloudy, aft. and ev. clear. |
| 2 | 30.126 | .317 | 24.0 | 8 | 25.0 | S.W. | do. do. do. |
| 3 | 30.217 | .162 | 25.3 | 20 | 27.3 | (Var.) | M. and aft. cloudy, ev. clear. |
| 4 | 30.059 | .047 | 38.2 | 15 | 36.0 | S.W. | Clear. |
| 5 | 30.001 | .058 | 39.3 | 14 | 36.0 | S. W. | Cloudy. *Rain* 7 P.M. commenced. |
| 6 | 29.902 | .245 | 40.0 | 14 | 36.7 | N.W. | do. do. stopped at 11 P.M. |
| 7 | 30.143 | .195 | 27.5 | 6 | 27.8 | W. | do. |
| 8 | 29.869 | .094 | 26.8 | 5 | 26.7 | N. W. | M. and aft. cloudy, ev. clear. |
| 9 | 30.083 | .113 | 24.0 | 9½ | 27.0 | (Var.) | Cloudy. |
| 10 | 30.040 | .125 | 34.7 | 18 | 26.0 | S.W. | do. |
| 11 | 29.987 | .205 | 37.3 | 10 | 34.3 | N.E. | do. *R*. 9 to 11 & 6½ P.M. all night |
| 12 | 29.462 | .160 | 55.0 | 19 | 58.0 | (Var.) | M. & af. cl'dy, ev. clear; *R* until 7 |
| 13 | 29.559 | .081 | 45.0 | 12 | 40.7 | S.W. | Cloudy, [P.M. strong wind. |
| 14 | 29.939 | .225 | 29.3 | 14 | 29.3 | W. | M. clear, aft. and ev. cloudy. |
| 15 | 30.164 | .048 | 31.3 | 11 | 35.0 | S.W. | do. do. |
| 16 | 29.895 | .128 | 45.3 | 18 | 44.0 | S.W. | Cloudy, 10 P.M. *R*. cont. all night. |
| 17 | 30.019 | .273 | 40.0 | 5 | 37.7 | N.W. | do. *R*. till 10 A. M., then *Sn*. & *R*. till 11¼ A. M. |
| 18 | 30.099 | .090 | 33.0 | 3 | 30.7 | N.E. | do. *S*.10A.M. to 3P.M. & 5to6P.M. |
| 19 | 30.151 | .127 | 32.3 | 8 | 30.3 | N.W. | do.7P.M.*Sn*.*Sl*.*R*.stop.dur.n'gt. |
| 20 | 29.656 | .300 | 38.3 | 10 | 35.7 | (Var.) | do.12M.fog aft.&ev. *R*. at night. |
| 21 | 29.640 | .511 | 43.0 | 32 | 31.0 | (Var.) | M. and aft. cloudy, ev. clear. |
| 22 | 30.303 | .078 | 22.3 | 9 | 25.0 | W. | Clear. |
| 23 | 30.267 | .292 | 20.5 | 9 | 21.0 | W. | do. 9 A. M. *Snow* about 10 min. |
| 24 | 30.242 | .225 | 24.3 | 19½ | 31.0 | S.W. | do. |
| 25 | 30.402 | .061 | 24.7 | 12 | 28.0 | E.N.E. | do. M. & aft. ev. cl'y; *Sn*. 8½ to |
| 26 | 29.821 | .150 | 43.0 | 22 | 43.7 | S.W. | Cloudy. [9½ P. M. |
| 27 | 29.788 | .136 | 35.8 | 10 | 27.0 | W. | Morn. and aft. cloudy, ev. clear. |
| 28 | 30.292 | .185 | 21.8 | 6 | 24.0 | N.W. | do. do. do. |
| 29 | 30.430 | .070 | 22.0 | 10 | 25.0 | N.E. | Cloudy. |
| 30 | 30.181 | .255 | 30.3 | 10 | 30.7 | N.E. | Morn. and aft. cloudy, ev. clear. |
| 31 | 29.733 | .247 | 36.7 | 11 | 26.0 | S.W. | do. do. do. |
| Monthly Mean | 30.008 | | 32.89 | | 31.82 | SW. NW. W. | Rain and melted snow during the month 2.32 inches. |
| High. | 30.471 | on 29th | 58° on 12th. | | 58° on 12th. | | |
| Low. | 29.384 | on 12th | 11½° on 24th | | 21° on 23d. | | |
| range. | 1.087 inch. | | 46½° | | 37° | | |

